# A Correlation Study on In Vitro Physiological Activities of Soybean Cultivars, 19 Individual Isoflavone Derivatives, and Genetic Characteristics

**DOI:** 10.3390/antiox10122027

**Published:** 2021-12-20

**Authors:** Han-Na Chu, Su-Ji Lee, Xiaohan Wang, Sang-Hoon Lee, Hye-Myeong Yoon, Yu-Jin Hwang, Eun-Suk Jung, Yongseok Kwon, Chi-Do Wee, Kyeong-A Jang, Haeng-Ran Kim

**Affiliations:** 1 Department of Agro-Food Resources, National Institute of Agricultural Sciences, Wanju 55365, Korea; hannachu@korea.kr (H.-N.C.); sujiseven@naver.com (S.-J.L.); spprigan@korea.kr (S.-H.L.); yjhwang1022@korea.kr (Y.-J.H.); haha1004@korea.kr (E.-S.J.); selenium2012@korea.kr (Y.K.); cdwee@korea.kr (C.-D.W.); jka1213@korea.kr (K.-A.J.); 2 National Agrobiodiversity Center, National Institute of Agricultural Sciences, Jeonju 54874, Korea; a649987318@korea.kr (X.W.); mmihm@korea.kr (H.-M.Y.)

**Keywords:** antioxidants, isoflavone derivatives, soybean, estrogen activity, correlation study, PLS-R, GWAS

## Abstract

The functionality of soybeans is an important factor in the selection and utilization of excellent soybean cultivars, and isoflavones are representative functional substances in soybeans, which exhibit effects on antioxidants, estrogen activity, and cancer, and prevent cardiovascular diseases. This study analyzed ABTS, DPPH, estrogen, ER (ER) alpha, UCP-1, and NO inhibition activities in 48 types of soybean cultivars, as well as the relationship with 19 isolated types of individual isoflavone derivatives. Statistical analysis was conducted to find individual isoflavone derivatives affecting physiological activities, revealing the high correlation of three types of derivatives: genistein 7-O-(6″-O-acetyl)glucoside (6″-O-acetylgenistin), genistein 7-O-(2″-O-apiosyl)glucoside, and glycitein. Based on these results, 15 types of soybean cultivars were selected (one control type, seven yellow types, six black types, and one green type), which have both high physiological activities and a high content of individual isoflavone derivatives. In addition, these high correlations were further verified through a genome-wide association study (GWAS) to determine the association between activities, substances, and genetic characteristics. This study comprehensively describes the relationship between the specific physiological activities of soybean resources, individual isoflavone derivative substances, and SNPs, which will be utilized for in-depth research, such as selection of excellent soybean resources with specific physiological activities.

## 1. Introduction

Soybean (*Glycine max* L.) is one of the world’s five major crops and a major source of vegetable protein and fat, and there has been increasing demand for its use in functional materials, cosmetics, and biofuels in recent years [1,2]. In East Asia, such as China, Japan, and South Korea, soybean has been consumed since ancient times in various forms, including soy sauce, soy milk, tofu, and soybean oil [3].

Soybeans contain lysine, an essential amino acid, as well as other various functional substances, such as dietary fiber, isoflavones, tocopherols, saponins, and phenolic acids [1,4]. Furthermore, approximately 12 isoflavones are known as representative functional substances: aglycones (daidzein, genistein, and glycitein), β-glycosides (daidzin, genistin, and glycitin), 6″-O-malonylglycoside (malonyldaidzin, malonylgenistin, and malonylglycitin), and 6″-O-acetlyglycosides (acetyldaidzin, acetylgenistin, and acetylglycitin), which are classified depending on their structure. Isoflavone was reported to show content changes according to various conditions, such as soybean variety, region, harvest time, soil fertility, light treatment, and storage duration [4]. The color and weight of soybeans were reported to affect the content of isoflavones, anthocyanins, and phenolic compounds, which, further, have different effects on antioxidant activity of compounds such as 2,2′-azino-bis-3-ethylbenzo-thiazoline-6-sulfonic acid (ABTS), 1,1-diphenyl-2-picrylhydrazyl (DPPH), and ferric reducing antioxidant power (FRAP). In addition, many studies have revealed that polyphenols, such as anthocyanins, isoflavones, and phenolic acids, are related to the health functional properties of soybeans, and that these compounds have individual or synergistic effects in cancer, diabetes, anti-inflammatory activity, and cardiovascular diseases [1,2].

Studies on the physiological activity of soybeans have been done in many countries. Regular consumption of soy-related products has been reported to increase bone density and prevent hypoestrogenic symptoms in menopausal women [5], and to further reduce the frequency and intensity of hot flashes, one of the menopause symptoms [6]. In addition, anti-inflammatory effects, such as inhibition of prostaglandin 2 (PGE2), and IL-1β were confirmed in RAW264.7 cells for black soybean extract [7], and when black soybean extract was administered to a diet-induced obesity (DIO) mouse model, decreases in TNF-α and IL-6 were observed, which are anti-inflammatory markers [8]. In previous studies related to obesity, isoflavone components in soybeans lowered blood lipid levels, thereby decreasing the risk of obesity and cardiovascular diseases [9], and when boiled and peeled soybeans were administered to Wistar rats among soybean recipes, lower cholesterol and weight were observed [10].

The recent expansion of the functional food market has resulted in increased demand for excellent functional materials; accordingly, consumers are requiring more accurate information regarding the functionalities. Thus, each country is exerting efforts to select and nurture excellent soybean cultivars, and promoting research on functional characteristics, expanding from the existing research on yield, cultivation characteristics, and genetic characteristics. Nevertheless, most of these studies have been limited to a small number of samples, or focused mainly on single functional substances and single efficacy [1]. Moreover, although individual compounds (particularly acylated compounds), among functional substances, are highly stable to heat and light, showing more advantage for industrial applications [11], research to date has focused mainly on the overall physiological activities of soybean or isoflavone extracts, and there is a general lack of studies on the effect of individual isoflavone derivatives.

Therefore, as part of the research being conducted for the selection of high-quality soybean varieties at the National Agrobiodiversity Center, National Institute of Agricultural Sciences (NAS) in South Korea, this study elucidates the relationship between the physiological activities of the sample and the substance by utilizing various statistical techniques for comprehensive analysis, rather than simplistically evaluating a single efficacy or component of substances. In this respect, physiological activities, such as ABTS, DPPH, estrogen, estrogen receptor (ER) alpha, and uncoupling protein-1 (UCP-1) activity, and nitric oxide (NO) inhibition activity, were analyzed to statistically understand correlations with 19 types of individual isoflavone derivatives that were analyzed with the same samples. Furthermore, a genome-wide association study (GWAS) was performed on physiological activities and individual isoflavone derivatives to genetically verify the correlation.

## 2. Materials and Methods

### 2.1. Experimental Materials

Among a total of 23,199 types of soybeans owned by the National Agrobiodiversity Center, 1000 varieties with excellent agronomic traits and functional components were selected, from which a total of 48 cultivars (variety, landrace, breeding line) were selected. The 48 selected cultivars and two control cultivars were sown in an open field in June 2019 at the National Agrobiodiversity Center, located in Jungdong, Jeonju, and the seeds harvested in September were freeze-dried and pulverized to produce powder for preparing analysis samples [2,4]. A total of 48 cultivars were classified by seed color, and one yellow type (C1) and one black type (C2) were selected as control cultivars, which were subdivided into 19 yellow types (Y1–Y19), 25 black types (B1–B25), and four green–black types (G1–G4) (Table 1).

### 2.2. Preparation of Soybean Seed Extract

The extract rich in flavonoids for soybean materials was obtained according to the extraction method described in previous studies, and the final extract was obtained through concentration and freeze-drying processes. Extraction of isoflavones from freeze-dried soybean powder was performed using 96% ethanol at 80 °C for 8 h with the ethanol over soybean powder ratio of 10:1 [12].

### 2.3. Antioxidant Activity Assay

#### 2.3.1. ABTS Radical Scavenging Activity

ABTS radical scavenging activity was measured as follows: 7.4 mM 2,2′-azino-bis-3-ethylbenzo-thiazoline-6-sulfonic acid (ABTS, Sigma-Aldrich, St. Louis, USA) and 2.6 mM potassium persulfate (Sigma-Aldrich, St. Louis, MO, USA) were prepared, and stirred for 24 h to form ABTS cations. Subsequently, the reagent was allowed to react with the soybean extract at a final concentration of 500 µg/mL for 30 min, and then absorbance was measured at 734 nm [13]. Ascorbic acid diluents (0–1000 µg/mL) were used to draw a calibration curve, and the result was calculated as the ascorbic acid equivalent (mg) per g of freeze-dried soybean seed weight (mg AA eq/g) [1].

#### 2.3.2. DPPH Radical Scavenging Activity

DPPH radical scavenging activity was measured as follows: 0.2 mM 1,1-diphenyl-2-picrylhydrazyl (DPPH, Sigma-Aldrich, St. Louis, USA) was prepared in methanol and reacted with the soybean extract at a final concentration of 500 µg/mL. After 30 min, absorbance was measured at 520 nm [13]. Ascorbic acid diluents (0–1000 µg/mL) were used to draw a calibration curve, and the result was calculated as the ascorbic acid equivalent (mg) per g of freeze-dried soybean seed weight (mg AA eq/g) [1].

### 2.4. Cell Potency Assay

#### 2.4.1. Estrogen and ER Alpha Activity

To obtain the MCF-7 cell supernatant, the cells were centrifuged at 2–8 °C at 1000× *g* for 20 min, and the supernatant was immediately collected, and stored in a freezer at −20 °C until the experiment. According to the method presented by the estrogen and ER alpha ELISA kit, the standard and samples were transferred to the provided plate and incubated. Subsequently, biotin detection antibody working solution and horseradish peroxidase–streptavidin conjugate (SABC) working solution were added to the plate in sequence, for reaction, and 3,3′,5,5′-tetramethylbenzidine (TMB) substrate and stop solution were further added. Absorbance was measured at 450 nm to calculate the result values.

#### 2.4.2. Uncoupling Protein-1 (UCP-1) Activity

To obtain a C3H10T1/2 cell supernatant, the cells were centrifuged at 2–8 °C at 5000× *g* for 5 min, and then the supernatant was immediately collected and subjected to the freeze–thaw process twice to break the cell membrane. The samples were stored in a freezer at −20 °C until the experiment. According to the method presented by the UCP-1 ELISA kit, the standard and samples were transferred to the provided plate and incubated. Subsequently, biotin detection antibody working solution and SABC working solution were added in sequence for reaction, and TMB substrate and stop solution were added. Absorbance was measured at 540 nm to calculate the result values.

#### 2.4.3. Nitric Oxide (NO) Inhibition Activity

RAW 264.7 cells, which were cultured at a concentration of 5 × 10^5^ cells/mL, were treated with LPS and soy extract for 24 h, and the secreted amount of NO was measured by using Griess reagent (G2930, Promega, Madison, WI, USA). Subsequently, the Griess reagent and the supernatant medium of the sample-treated cells were mixed at the ratio of 1:1:1 and reacted in the dark for 10 min. Absorbance was measured at 520 nm [14].

### 2.5. Characterization of Isoflavone in Soybean Core Genetic Resource Seeds

#### 2.5.1. Isoflavone Extraction and Solid Phase Extraction (SPE) Process

Ten milliliters of isoflavone extraction solvent (methanol:distilled water:formic acid = 50:45:5, *v*/*v*/*v*) and 1 g powder sample were mixed, stirred for 5 min, and centrifuged at 3600 rpm for 15 min (Gyrozen Co., Daejeon, Korea), and the supernatant was filtered. For the SPE process, 2 mL methanol and 5 mL water were sequentially flowed for activation. Each 5 mL of the filtrate and the internal standard material (6-methoxyflavone, 50 ppm) were passed through an SPE cartridge (Heper Sep C18, Thermo Fisher Scientific, Waltham, MA USA) and washed with water, and the isoflavones attached to the column were eluted with methanol. The isoflavone extract was evaporated under nitrogen gas, filtered through a 0.2 µm filter, and analyzed by UPLC-DAD-QToF/MS [4].

#### 2.5.2. Isolation and Component Analysis of Individual Isoflavone Derivatives

A CORTECS UPLC T3 (2.1 × 150 mm I.D., 1.6 µm; Waters, Wexford, Ireland) analytical column and CORTECS UPLC T3 VanGuardTM (2.1 × 50 mm I.D., 1.6 µm; Waters) guard column were utilized for separation of individual isoflavones from soybean seeds. In addition, an ultra-performance liquid chromatography system equipped with a diode array detector (Waters Co., Miliford, MA, USA) and a quadrupole time of flight mass spectrometer (UPLC-DAD-QToF/MS, Waters Micromass, Manchester, UK) were used. The individual isoflavone components were analyzed and detected by diode array detector and mass detector simultaneously, and their retention time differed by 0.1 min. The UV detector detects the components first, then the MS detector detects 0.1 min later. The detection wavelength, sample injection volume, and flow rate were set to 210–400 nm (representative wavelength, 254 nm), 1 µL, and 0.3 mL/min, respectively. To separate individual isoflavones, water containing 0.5% formic acid, and acetonitrile containing 0.5% formic acid, were utilized as solvents A and B, respectively, for a mobile phase. The mobile phase of eluent B was 5% at the starting point, and raised to 25%, 50%, and 90% up to 20 min, 25 min, and 30 min, respectively, which was maintained up to 32 min, and further lowered to 5% up to 35 min, and then maintained and stabilized at 5% up to 40 min. A chemical library was developed by compiling reports obtained from published literature where the data are confirmed by LC/MS and NMR analysis. This library was used as a reference to determine the presence of the compounds in soybean, elution order of the isomers, mass fragmentation pattern, and UV absorbance characteristics of identified compounds. Moreover, authentic standards were used for confirmation of results wherever available. As a corresponding optimization condition, the voltages of the capillary, extraction cone, and sampling cone were set to 3500 V, 4.0 V, and 40 V, respectively, and the ion source temperature and desolvation temperatures were set to 120 °C and 500 °C, respectively. In addition, desolvation and cone gas were set to 1050 and 50 L/h, respectively, and the scan range of mass was set to *m*/*z* 200 to 1200. Individual isoflavone derivative components were primarily analyzed and further identified by referring to mass fragment ion pattern information from the previously prepared soybean library. Quantification of individual isoflavones was performed by comparing the area of each component at a 1:1 ratio compared to the internal standard material (6-methoxyflavone) injected during pretreatment, to calculate a relative quantitative value (mg/100 g dry weight) [4].

### 2.6. DNA Extraction and Genotyping of Soybean Seeds

The whole grain soybean tissue was sampled. Total DNA was extracted using the Bead™ Genomic DNA Prep Kit for plants (Biofact, KOR) according to the manufacturer’s instructions. Genomic DNA was quantified using a UVS-99 UVISDrop Spectrophotometer (ACTGene, Piscataway, NJ, USA), the ratio of A260/A280 nm was determined, and DNA was diluted with water to 100 ng/μL. DNA quality was verified on 1% agarose gel, and DNA stored at −20 °C. The 180 K AXIOM^®^ SoyaSNP array [15] was used to genotype 48 soybean accessions. We carried out genotyping and quality control procedures according to the Axiom^®^ Genotyping Solution Data Analysis User Guide and Axiom Best Practice Supplement (http://www.affymetrix.com/; last accessed 3 November 2021). The default dish quality control (DQC) threshold value was 0.82 for all Axiom arrays except Axiom_BOS1 which was 0.95 (DQC by Array Power Tools version 1.18 in the analysis workflow). The DQC value of all samples was >0.82, and the QC call rate value was >97%. Excluding 586 single-nucleotide polymorphisms (SNPs) not included on 20 chromosomes, 180,375 SNPs were retained for the GWAS. On average, approximately 1 SNP is provided for every 6.1 kb sequence.

### 2.7. Statistical Analysis

Data were represented as means±standard deviation (SD). The experiment was repeated three times to significantly verify the difference between the measured values, and the significance between the control group and the experimental group was confirmed by IBM SPSS Ver. 26.0 (SPSS Inc., Chicago, IL, USA). A one-way analysis of variance (ANOVA) was performed by utilizing IBM SPSS software to verify the difference between samples.

In order to examine the correlation between the physiological activities of soybean cultivars, and individual isoflavone derivatives, a correlation heat map was made using R. After this verification, partial least squares regression (PLSR) was performed by utilizing XLSTAT (2021.3.1 Trial Version, Addinsoft, Paris, France).

A GWAS was conducted to examine the relationship between the physiological activities of soybean cultivars, individual isoflavone derivative-related genes, and molecular regulation mechanisms between traits. Six GWAS models were used: general linear model (GLM), mixed linear model (MLM), compressed mixed linear model (CMLM), multiple loci mixed model (MLMM) fixed and random model circulating probability unification (FarmCPU), and Bayesian information and linkage disequilibrium iteratively nested keyway (BLINK) implemented in the genome association and prediction integrated tool (GAPIT) R package. In GAPIT3, the Bonferroni multiple test threshold was used to determine significance, scilicet calculated from the formula of –log (0.05/effective number of SNPs).

## 3. Results and Discussion

### 3.1. Physiological Activity Analysis of Soybean Seeds

#### 3.1.1. Antioxidant Activity of Soybean Seeds (Radical Scavenging Activity)

As a result of examining the ABTS and DPPH activities of soybean cultivars (Table 2), the ABTS activity of all sample was in the range of 1.60–11.93 mg AA eq/g, and the average was 4.17 mg AA eq/g. By color, the activity was determined in the order of black group (4.79 mg AA eq/g) > yellow group (3.48 mg AA eq/g) > green group (3.17 mg AA eq/g). The sample with the highest activity was the black B17 sample (11.93 ± 1.08 mg AA eq/g), with 2.4 times higher activity than the black control, C2.

For DPPH activity, the total sample range was from 0.28–6.20 mg AA eq/g, with an average of 1.85 mg AA eq/g. By color of soybeans, the DPPH activity, as with ABTS, was determined to be high in the order of black group (2.05 mg AA eq/g) > yellow group (1.64 mg AA eq/g) > green group (1.54 mg AA eq/g). Previous studies have shown that black soybeans have high ABTS and DPPH activities because black soybean contains a large amount of anthocyanin and isoflavone, thereby confirming a difference in antioxidant activity depending on seed color [16,17]. Proanthocyanidin, contained in black soybean and abundant in various foods, is a polyphenolic substance of anthocyadin and is a secondary metabolite of food synthesized through the biosynthesis pathway of flavonoid, exhibiting antibacterial, anti-inflammatory, and antiallergic properties in addition to antioxidant activity [18]. In a study on sweet potatoes, purple sweet potatoes with high anthocyanin content showed higher antioxidant and antidiabetic effects [11]. The sample with the highest DPPH activity, as with ABTS activity, was the B17 sample (6.20 ± 0.35 mg AA eq/g), with 4.8 times higher activity than the black control, C2. The results suggest that the black B17 sample had a significantly high antioxidant activity compared to other soybean samples, and that it would have a high antioxidant potential as a landrace that has not been bred, as shown in the sample information (Table 1).

#### 3.1.2. Estrogen and ER Alpha Activity in MCF-7 Cells

Estrogen is a female sex hormone and a steroid-type hormone that is directly involved in the menstrual cycle, pregnancy, and menopause to control various functions [19]. Imbalance of hormones, such as estrogen, may result in diseases, including obesity, osteoporosis, diabetes, hypertension, hyperlipidemia, and metabolic syndrome, and isoflavones, which are abundant in soybeans, are phytoestrogens used as potential replacement therapies for hormone-dependent diseases [20].

**Figure 1:**
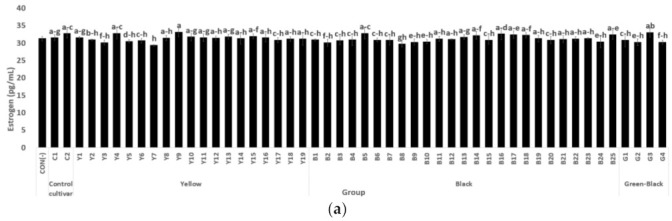

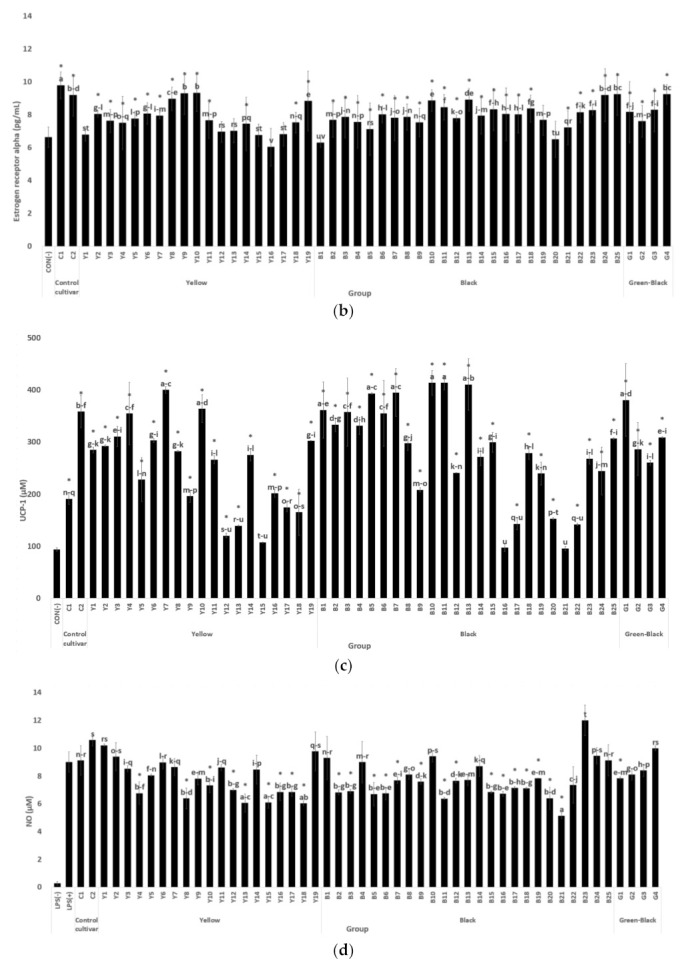
Comparison of estrogen activity (**a**), estrogen receptor alpha activity (**b**), UCP-1 activity (**c**), and NO inhibition activity (**d**) by soybean seed coat color. ^a–v^ Means followed by the same letter within rows are significantly different at *p* < 0.05, Duncan’s multiple range test. * Significantly different from CON and LPS(+) at *p* < 0.05.

The estrogen activity of all samples ranged from 29.40–33.16 pg/mL, and the average was 31.31 pg/mL. By the color of soybeans, the estrogen activity was determined to be high in the order of yellow group (31.35 pg/mL) > black group (31.24 pg/mL) > green group (31.12 pg/mL). Furthermore, most of the soybean samples, including the control, showed an increasing tendency in comparison to CON(-), while there was no difference between the samples (Figure 1a).

The ER alpha activity ranged from 6.03–9.79 pg/mL, with an average of 7.95 pg/mL. The ER alpha activity was determined to be high in the order of green group (8.32 pg/mL), black group (7.95 pg/mL), and yellow group (7.70 pg/mL). Most of the soybean samples, including the control, showed a significantly high activity in comparison to CON(-), and there was a significant difference between the samples. Meanwhile, the yellow bean control sample C1 showed significantly higher activity than the yellow sample Y1-19, and the black control sample C2 also showed high activity, similar to that of B24 and 25 among the black samples (B1–B25) (Figure 1b).

Estrogen increases cell proliferation by binding to ER and stimulating receptor-mediated signaling pathways in breast cancer cells. The physiological effects of estrogen are mainly mediated by ER alpha, and isoflavone can exert estrogen effects through ER binding because the chemical structure of isoflavone is similar to that of estrogen-17β estradiol [21]. Thus, estrogen and ER alpha activities showed different tendencies in this experiment. As estrogen is a hormone, it was regulated by various metabolic mechanisms and it was considered that isoflavone had less of an effect on estrogen than ER.

#### 3.1.3. Antiobesity Activity Based on Brown Fat Transformation Activity (UCP-1 Activity of C3H10T1/2 Cells)

Body fat mainly consists of white fat. White fat is used as an energy source for the body in emergency situations, while an excess of white fat could induce obesity and diabetes [22]. In contrast, brown fat is known to promote metabolism by generating heat, and to prevent diseases, such as obesity, diabetes, heart disease, and cancer, by consuming stored energy as heat. White fat can be changed to beige/brown adipocytes due to browning by environmental stimuli (including cold temperatures and spicy food intake) [23,24]. Beige/brown fat can improve obesity and metabolic diseases induced by the accumulation of white fat, as well as helping improve body metabolism [24].

Thus, this study evaluated the antiobesity activity of soybean seeds by investigating the UCP-1 gene expression, which is an indicator of brown fat activity in adipocytes (Figure 1c). The UCP-1 activity of all soybean samples ranged from 95.85–413.85 µM, with an average of 272.26 µM. The UCP-1 brown fat transformation activity was high in the order of green group (309.20 µM) > black group (282.17 µM) > yellow group (251.14 µM). This represents a high degree of brown fat conversion activity. The sample with the highest activity was B11 with a black color at 413.85 µM, which was 1.2 times higher than that of the black control, C2 (358.61 µM). The results suggest that the black B11 sample had a significantly high brown fat transformation activity compared to other soybean samples, and that it would have a high antiobesity potential as a landrace that has not been bred, as shown in the sample information (Table 1).

#### 3.1.4. NO Production Inhibition Activity of RAW 264.7 Cells

Inflammatory response is a defense response to protect the body from damage induced by infection by pathogens, or physical or chemical stimuli. An appropriate inflammatory response helps the body to maintain homeostasis, whereas an excessive defensive response and continuous inflammatory response can induce asthma, atopic dermatitis, and neurodegenerative diseases [25]. Studies have reported that NO, one of the indicators of inflammatory response, plays an important role in the mechanism of oxidative damage in the human body, and that the inhibition of NO production helps anti-inflammatory activity. The NO production increase after injecting LPS into RAW264.7 cells was attributed to inhibition of NO production due to treatment with black soybean seed extract [26].

According to the results of analyzing the NO production inhibition activity of soybean seeds, the NO production inhibition activity of all soybean samples ranged from 5.14–11.99 µM, with an average of 7.91 µM. The black group (7.75 µM) showed a low activity, indicating inhibition of inflammatory mediator generation, and the anti-inflammatory activity was determined to be high in the order yellow group (7.77 µM) then green group (8.57 µM). The sample with the highest activity was the black soybean sample B21 with 5.14 µM, with 2.1 times higher inhibition activity than the black control, C2 (10.58 µM). Among yellow soybeans, the Y18 sample showed the highest inhibition activity, at 6.02 µM, whose inhibition activity was 1.5 times higher than that of the yellow sample control, C1 (9.11 µM). These samples would also have a high anti- inflammatory potential as a landrace in selecting soybean cultivars as anti-inflammatory materials (Figure 1d).

### 3.2. Composition and Content of Isoflavone in Soybean Seed Core Genetic Resources

**Figure 2:**
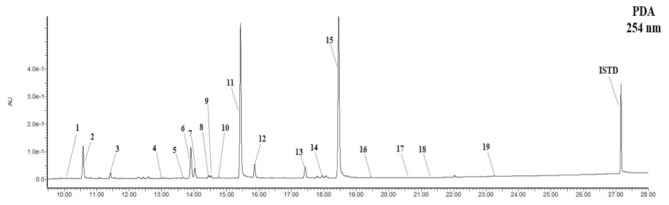
UPLC-DAD chromatograms of isoflavones in soybean (*Glycine max* L.) seeds at 254 nm: (1) genistein 5-O-glucoside, (2) daidzein 7-O-glucoside (daidzin), (3) glycitein 7-O-glucoside (glycitin), (4) genistein 7-O-(6″-O-apiosyl)glucoside, (5) genistein 7-O-(2″-O-apiosyl)glucoside (ambocin), (6) genistein 7-O-glucoside (genistin), (7) daidzein 4′-O-(6″-O-malonyl)glucoside, (8) genistein 5-O-(6″-O-malonyl)glucoside, (9) daidzein 7-O-(4″-O-malonyldaidzin), (10) glycitein 4′-O-(6″-O-malonyl)glucoside, (11) daidzein 7-O-(6″-O-malonyl)glucoside (6″-O-malonyldaidzin), (12) glycitein 7-O-(6″-O-malonyl)glucoside (6″-O-malonylglycitin), (13) genistein 4′-O-(6″-O-malonyl)glucoside, (14) genistein 7-O-(4″-O-malonyl)glucoside (4″-O-malonylgenistin), (15) genistein 7-O-(6″-O-malonyl)glucoside (6″-O-malonylgenistin), (16) daidzein, (17) glycitein, (18) genistein 7-O-(6″-O-acetyl)glucoside (6″-O-acetylgenistin), (19) genistein, internal standard (ISTD): 6-methoxyflavone 50 ppm.

According to the result of identifying isoflavones isolated from soybean seeds, 19 types of individual isoflavone derivative (10 types of genistein, five types of daidzein, and four types of glycitein) components were identified at UV = 254 nm (Figure 2). Previous studies, which were conducted as part of joint research for the selection of excellent soybean cultivars, have revealed various glycoside forms, including glucose bonds at the 7-OH, 5-OH, or 4′-OH position of the aglycone, and, additionally, malonic or acetic acid bonds to these structures, in addition to the aglycone structures of daidzein, genistein, and glycitein [3]. In addition, the previous studies have provided information on the composition and content of 19 types of isoflavones, including newly estimated components, plus 12 core components of soybeans. They identified genistein 7-O-(2″-O-apiosyl)glucoside and genistein 7-O-(6″-O-apiosyl)glucoside(ambocin) as novel glycosides in soybeans, and for the first time identified glycitein 4′-O-(6″-O-malonyl)glucoside among malonyl-glucoside [4].

The total isoflavone content of all soybean samples was in the range of 95.96–490.56 mg/100 g in dry weight, and B7 (490.56 mg/100 g, DW) indicated the highest content, followed by Y13 (467.69 mg/ 100 g, DW), Y11 (451.55 mg/100 g, DW), B1 (445.21 mg/100 g, DW), and B20 (409.33 mg/100 g, DW) [4]. The sample with a high total isoflavonoid content is the Uram soybean variety, Y13, and the remaining soybean samples would have a high potential as a landrace in selecting soybean cultivars.

### 3.3. Correlation Analysis between Physiological Activities and Components of Soybean Samples

**Figure 3:**
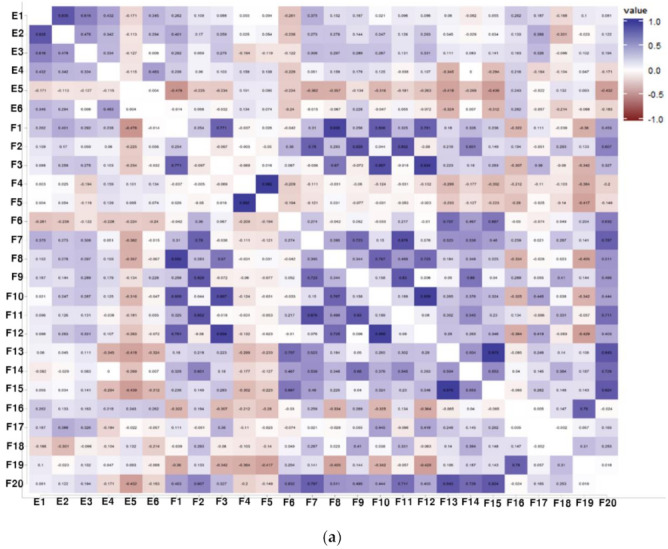

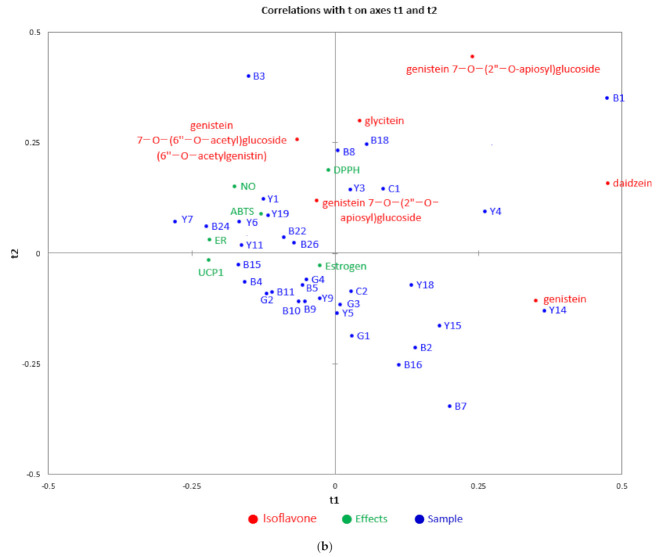
(**a**) Correlation analysis of effects and isoflavone with soybean seed (E1, ABTS; E2, DPPH; E3, estrogen; E4, estrogen receptor alpha; E5, UCP-1; E6, NO; F1, genistein 5-O-glucoside; F2, daidzein 7-O-glucoside (daidzin); F3, glycitein 7-O-glucoside (glycitin); F4, genistein 7-O-(6″-O-apiosyl)glucoside; F5, genistein 7-O-(2″-O-apiosyl)glucoside; F6, genistein 7-O-(6″-O-apiosyl)glucoside; F7, daidzein 4′-O-(6″-O-malonyl)glucoside; F8, genistein 5-O-(6″-O-malonyl)glucoside; F9, daidzein 7-O-(4″-O-malonyl)glucoside (4″-O-malonyldaidzin); F10, glycitein 4′-O-(6″-O-malonyl)glucoside; F11, daidzein 7-O-(6″-O-malonyl)glucoside (6″-O-malonyldaidzin); F12, glycitein 7-O-(6″-O-malonyl)glucoside (6″-O-malonylglycitin); F13, genistein 4′-O-(6″-O-malonyl)glucoside; F14, genistein 7-O-(4″-O-malonyl)glucoside (4″-O-malonylgenistin); F15, genistein 7-O-(6″-O-malonyl)glucoside (6″-O-malonylgenistin); F16, daidzein; F17, glycitein; F18, genistein 7-O-(6″-O-acetyl)glucoside (6″-O-acetylgenistin); F19, genistein; F20, total flavonoid). (**b**) Analysis of isoflavones, effects, and samples by PLSR. ABTS (radical scavenging activity), DPPH (radical scavenging activity); estrogen (estrogen activity); ER-α (estrogen receptor alpha activity); UCP-1 (Uncoupling protein-1 activity); NO (nitric oxide inhibition activity). Control cultivar (C1–C2); yellow group (Y1–Y19); black group (B1–B25); green group (G1–G4).

The correlations between the six types of physiological activities of soybean samples, and components of individual isoflavone derivatives, were determined by Pearson’s correlation coefficients and are presented as a heat map (Figure 3a). ABTS (E1) and DPPH (E2) showed a high correlation, with a coefficient of 0.835, in the relationship between activities and activities, and ABTS (E1) and estrogen (E4) indicated a correlation coefficient of 0.616. Antiobesity activity based brown fat transformation activity, which was analyzed through UCP-1 activity, showed insignificant correlation with other activities. This insignificance could be attributed to the difference from various mechanisms of obesity, because this study included an analysis with brown fat transformation activity, a recently introduced indicator, in contrast to the studies reporting that other activities, such as antioxidant activity, have an effect on antiobesity as well.

An investigation of correlations between the 19 types of individual isoflavone derivatives (20 types, including total flavonoids) mostly shows a positive correlation. Noticeably, genistein 7-O-(6″-O-apiosyl)glucoside (F4) showed a significantly high positive correlation with genistein 7-O-(2″-O-apiosyl)glucoside (F5), having a correlation coefficient of 0.982, whereas its correlations with the other 18 substances were determined to be insignificant. In addition, the correlation coefficient between genistein 4′-O-(6″-O-malonyl)glucoside (F13) and genistein 7-O-(6″-O-malonyl)glucoside (6″-O-malonylgenistin) (F15) was 0.975 and the correlation coefficient between glycitein 4′-O-(6″-O-malonyl)glucoside (F10) and glycitein 7-O-(6″-O-malonyl)glucoside (6″-O-malonylglycitin) (F12) was 0.959.

In the correlation between physiological activities and components of individual isoflavone derivatives, DPPH (E2) and genistein 5-O-glucoside (F1) indicated a positive correlation with the coefficient of 0.401 (Figure 3). Furthermore, the correlation between activities and activities was insignificant for UCP-1 (E5), which showed a negative correlation with genistein 5-O-glucoside (F1), having a coefficient of −0.475. Lee et al. (2020) identified genistein 5-O-glucoside as a novel component in which glucose is bound to the 5-OH position, rather than the existing 7-OH position. As genistein 5-O-glucoside was determined to have a correlation with DPPH, functional verification of new components can be performed through antioxidant studies, such as radical scavenging activity and antiobesity studies.

Partial least squares regression (PLSR) analysis was used with the activity analysis results of soybean seeds, and the content of individual isoflavone derivative components. The PLSR is an efficient statistical method, which utilizes the PLS technique to estimate parameters determining connection among variables, and further considers multicollinearity, originating from typical regression analysis, to simultaneously add many independent variables [27]. Thus, a statistical model was created from the results of various experiments on soybean seeds to increase the interpretation power.

The results of PLSR analysis were presented on the activities of ABTS, DPPH, estrogen, ER alpha, UCP-1, and NO. The relationships between 48 types of soybean cultivars (50 types, including control samples), six types of activities, and 19 types of individual isoflavone derivative components (20 types, including total flavonoids) were analyzed, and the results were summarized, excluding samples and substances with insignificant correlations (Figure 3b). Although all six types of activities were located in the negative direction with respect to t1, there were high correlations, in particular, in ABTS activity, ER alpha activity, UCP-1 activity, and NO inhibition activity due to its proximal location. In contrast, DPPH and estrogen activities were located at a distance from the remaining activities. Meanwhile, the three types of individual isoflavone derivatives that were closest to these activities are genistein 7-O-(2″-O-apiosyl)glucoside, genistein 7-O-(6″-O-acetyl)glucoside (6″-O-acetylgenistin), and glycitein, showing a high correlation with activities. In particular, the DPPH activity indicated significantly high correlations with these three types of derivatives, having the closest proximity. Jang et al. (2019) isolated and purified 12 individual derivatives from purple sweet potatoes, and verified that peak number 9 had antioxidant and antidiabetic effects through both in vitro and in vivo experiments [11]. However, because a trace amount of sample can be secured in the case of isolating and purifying individual derivatives, there is a limitation in data acquisition. Moreover, this technique can be applied to single component and single efficacy cases only. Thus, the application of this statistical technique would be appropriate to clarify the correlation from a comprehensive viewpoint.

Samples that are excellent in six types of activities, and correlated to the aforementioned three types of individual isoflavone derivatives, are as follows (Figure 3b). With four types of activities—ABTS activity, ER alpha activity, UCP-1 activity, and NO inhibition activity—Y1, Y6, Y7, Y11, and Y19 in the yellow group and B4, B15, and B24 in the black group show high correlations, indicating close proximity. The DPPH activity was highly correlated with B8 and B18 in the black group, Y3 in the yellow group, and C1 of the yellow control group. The estrogen activity showed high correlations with G4 in the green group, B5 in the black group, and Y9 in the yellow group.

**Figure 4:**
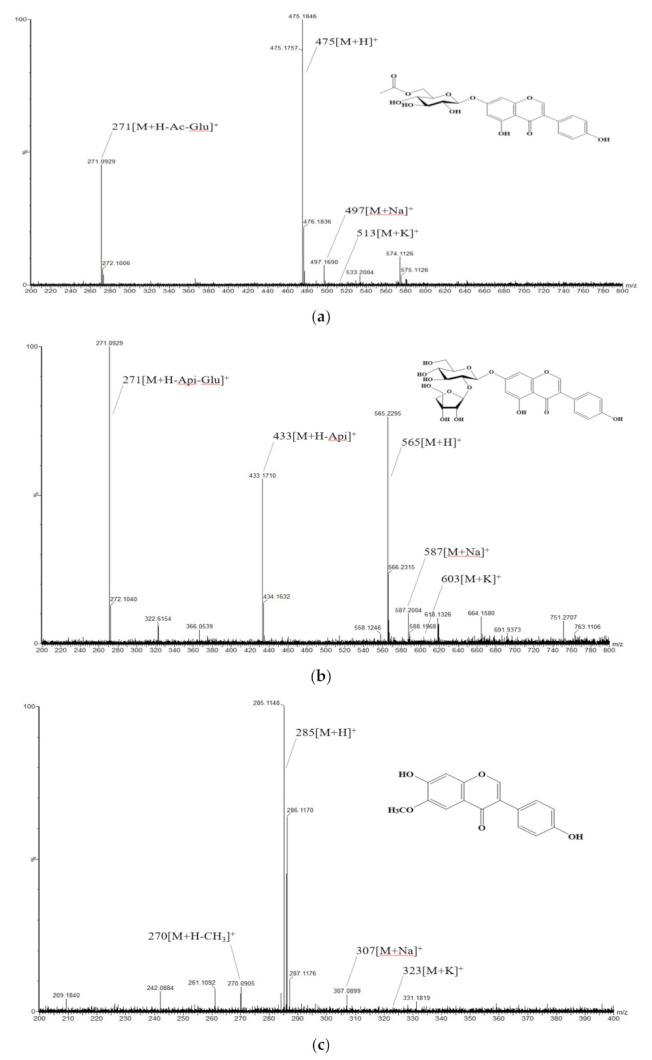
Positive mass fragmentations (*m*/*z*, [M+H]^+^) and structures of three representative isoflavones in soybean (*Glycine max* L.) seeds. (**a**) 6″-O-acetylgenistin (*m*/*z* 475), (**b**) genistein 7-O-(2″-O-apiosyl)glucoside (*m*/*z* 565), (**c**) glycitein (*m*/*z* 285).

Figure 4 presents the positive mass fragmentations (*m*/*z*, [M+H]^+^) and structures regarding the three types of individual isoflavone derivatives, which were highly correlated with the six types of activities according to the PLSR analysis results. Genistein 7-O-(2″-O-apiosyl)glucoside (*m*/*z* 565) has been reported as a novel glycoside of soybean in a previous study [4]. Further research is needed on how three types of derivatives affect the physiological activities.

### 3.4. Identification of the Relationship between Individual Isoflavone Derivatives Having High Correlation with Physiological Activities of Soybean Cultivars, and Genetic Characteristics

**Figure 5:**
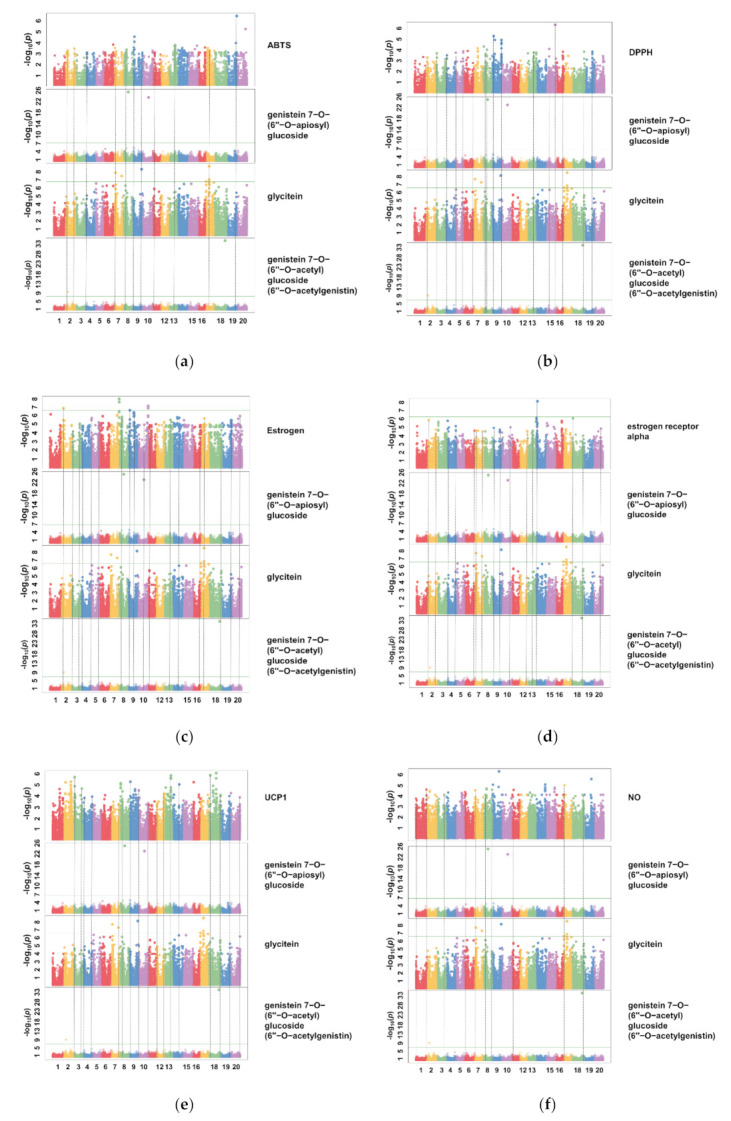
GWAS of 48 individual soybean isoflavone derivatives. Dashed lines present different p valued of SNPs associated with (**a**) ABTS (radical scavenging activity), (**b**) DPPH (radical scavenging activity), (**c**) estrogen (estrogen activity), (**d**) ER-α (estrogen receptor alpha activity), (**e**) UCP-1 (uncoupling protein-1 activity), (**f**) NO (nitric oxide inhibition activity), and three parameters (genistein 7-O-(6″-O-acetyl)glucoside(6″-O-acetylgenistin), genistein 7-O-(2″-O-apiosyl)glucoside, and glycitein). Significant associations were identified using criterion of −log10(P) > 3.

If the single-nucleotide polymorphism (SNP) of a crop is investigated, and an analysis via a GWAS is conducted, the correlation between the genetic variation and the trait of the sample can be identified. Initially, GWASs were developed to search for disease-causing genes and utilized in the medical field. GWASs have a higher resolution than linkage mapping, and can be used to explore natural populations, as well as interbreeding groups [28]. GWASs have been extensively used to locate genes associated with traits of interest in crops, improving the accuracy of associations between genetic markers, and natural population traits [29,30]. Thus, a GWAS analysis was conducted to investigate the genetic association of isoflavone derivatives, which have a high correlation with the activities of the aforementioned soybean cultivars (Figure 5).

As presented in the above method, using 180,375 SNPs, from Han et al.’s method [31] of confirming the role of a set of genes in different populations, in this study, when comparing SNPs that can be associated with different traits, we set the threshold to -log10(p)>3 to ensure sufficient data mining. Results showed that there were 107 out of 798 SNPs with ABTS activity (13.41%), 54 out of 474 SNPs with DPPH activity (11.39%), 175 out of 3459 SNPs with estrogen activity (5.06%), 461 out of 3283 SNPs with ER alpha activity (14.04%), 294 out of 2763 SNPs with UCP-1 activity (10.64%), and 265 out of 3780 SNPs with NO inhibition activity (7.01%) (Table 3). SNPs contained the three types of isoflavone derivatives, genistein 7-O-(2″-O-apiosyl)glucoside, genistein 7-O-(6″-O-acetyl)glucoside (6″-O-acetylgenistin), and glycitein, which were found to overlap. The overlapping of SNPs implies that the same SNP is present in the GWAS results of both traits, which suggests that there is a high likelihood of involvement of the same gene in the regulatory pathway for the physiological activities (ABTS activity, DPPH activity, estrogen activity, ER alpha activity, UCP-1 activity, and NO inhibition activity), and the three types of isoflavone derivatives (genistein 7-O-(2″-O-apiosyl)glucoside, genistein 7-O-(6″-O-acetyl)glucoside (6″-O-acetylgenistin), and glycitein).

Moreover, the chromosome position was highly related to the physiological activities of soybean cultivars of ABTS activity, DPPH activity, UCP-1 activity, and NO inhibition activity, which were located at chromosomes 19, 15, 13/18, and 9/19 (P, −log10(p) > 6.45), respectively, while estrogen activity and ER alpha activity were located at chromosomes 2/8/10 and 13 (P, −log10(p) > 6), respectively. These results suggest that by the high probability that, in the case of the six types of physiological activities of soybean cultivars, the gene (or gene of the chromosome) located on the chromosome is highly likely to affect the expression level of the activities.

## 4. Conclusions

In this study, 48 types of core soybean cultivars were grown, selected by the National Agrobiodiversity Center, and classified into three types of seed colors (yellow 19, black 25, and green 4), and two control cultivars (yellow and black) for analysis. This study identified physiological activities—antioxidant activity (ABTS, DPPH), menopausal improvement effect (estrogen, ER alpha), antiobesity effect (UCP-1 activity), and anti-inflammatory effect (NO inhibition ability)—and further analyzed the relationships with 19 types of individual isoflavone derivatives, isolated and identified from the same samples. In addition, genotyping was performed to examine the relationship with the genetic characteristics.

According to the results of the correlation analysis regarding individual isoflavone derivatives affecting physiological activities, the three types of derivatives—genistein 7-O-(6″-O-acetyl)glucoside (6″-O-acetylgenistin), genistein 7-O-(2″-O-apiosyl)glucoside, and glycitein—showed a high correlation. Based on this result, we selected 15 types of soybean cultivars (one control type, seven yellow types, six black types, and one green type) with high physiological activities and high content of these individual isoflavone derivatives. In addition, this study has verified a high correlation through a GWAS to determine the correlation between activities, substances, and genetic characteristics. The locations of chromosomes highly related to the physiological activities of soybean cultivars were presented, predicting that these chromosomes (or genes of chromosomes) would affect the physiological activities of soybean cultivars. This study comprehensively describes the relationship between specific physiological activities of soybean cultivars, individual isoflavone derivative substances, and SNPs, which will be utilized in more in-depth research, such as selection of excellent soybean resources with specific activities. Moreover, because most of the soybean samples with distinct functionally superior characteristics are landraces, they could be used for the development of highly functional varieties and functional foods in the future.

## Figures and Tables

**Table 1:** Classification by different seed coat colors and information of soybean seeds.

Seed Coat Color	Code	Introduction Number	Name	Origin	Cultivar Type
Control	C1(Yellow)	IT 212859	Daewon	Korea	Control cultivar
	C2(Black)	IT 213192	Cheongja 2	Korea	Control cultivar
Yellow	Y1	IT 024099	YJ208-1	Korea	Landrace
	Y2	IT 104690	Kongnamul Kong	Korea	Landrace
	Y3	IT 113218	Kongnamul Kong	Korea	Landrace
	Y4	IT 153844	KLS 87248	Korea	Landrace
	Y5	IT 155963	Nongrim 51	Japan	Variety
	Y6	IT 171080	PI 467319	China	Variety
	Y7	IT 195514	Jang Kong	Korea	Landrace
	Y8	IT 219581	Myungjunamul Kong	Korea	Variety
	Y9	IT 229418	Danmi 2	Korea	Variety
	Y10	IT 229421	Hoseo	Korea	Variety
	Y11	IT 231360	Kongnamul Kong	Korea	Landrace
	Y12	IT 263155	Sinpaldalkong 2	Korea	Variety
	Y13	IT 263167	Uram	Korea	Variety
	Y14	IT 263852	Chungnamyeongi-1997-3	Korea	Landrace
	Y15	IT 269982	Milyang 247	Korea	Breeding line
	Y16	IT 270002	Jungmo 3008	Korea	Variety
	Y17	IT 274571	GNU-2007-14613	Korea	Landrace
	Y18	IT 274592	GNU-2007-14723	Korea	Landrace
	Y19	IT 324099	CS 00728	China	Breeding line
Black	B1	IT 021665	PI 90763	China	Landrace
	B2	IT 143347	KLS86185	Korea	Landrace
	B3	IT 161904	PI 84578	Korea	Landrace
	B4	IT 177271	Geomjeong Kong-5	Korea	Landrace
	B5	IT 177573	Geomjeong Kong-5	Korea	Landrace
	B6	IT 177709	Geomjeong Kong-4	Korea	Landrace
	B7	IT 178054	Geomjeong Kong-1	Korea	Landrace
	B8	IT 186183	Kongnamul Kong	Korea	Landrace
	B9	IT 189215	94yuja4	Korea	Landrace
	B10	IT 194560	Geomjeong Kong	Korea	Landrace
	B11	IT 212805	Chungnamseocheon-1999-98	Korea	Landrace
	B12	IT 224192	Jeonbukgunsansujip	Korea	Landrace
	B13	IT 228822	409	Korea	Landrace
	B14	IT 231544	Jwinuni Kong	Korea	Landrace
	B15	IT 239896	Jwineori Kong	Korea	Landrace
	B16	IT 252252	Neoljeokseoritae	Korea	Landrace
	B17	IT 252748	294	Korea	Landrace
	B18	IT 252768	326	Korea	Landrace
	B19	IT 263853	Geomen Kong	Korea	Landrace
	B20	IT 274515	GNU-2007-14502	Korea	Landrace
	B21	IT 275005	197	Korea	Landrace
	B22	IT 308619	Junyeori Kong	Korea	Landrace
	B23	IT 311261	Jwinuni Kong	Korea	Landrace
	B24	K 137773	Heugseong	Korea	Variety
	B25	IT 194558	Geomjeong Kong	Korea	Landrace
Green–Black	G1	IT 154351	KAS579-1	Korea	Landrace
	G2	IT 154724	KAS651-21	Korea	Landrace
	G3	IT 178160	Geomjeong Kong	Korea	Landrace
	G4	IT 186048	Gangwonyanggu-1994-3709	Korea	Landrace

**Table 2:** ABTS and DPPH radical scavenging activity in soybean seeds.

Seed Coat Color	Code	ABTS (mg AA eq/g)	DPPH (mg AA eq/g)
Control cultivar	C1	4.84 ± 1.1 ^e–i^	2.43 ± 0.52 ^b–i^
	C2	5.04 ± 1.32 ^d–h^	1.32 ± 0.50 ^d–j^
	Range	4.84–5.04	1.32–2.43
	Mean	4.94	1.88
Yellow	Y1	2.25 ± 0.83 ^m–o^	1.20 ± 0.14 ^d–j^
	Y2	1.92 ± 0.98 ^n–o^	0.99 ± 0.13 ^f–j^
	Y3	2.47 ± 1.05 ^l–o^	1.30 ± 0.05 ^d–j^
	Y4	3.24 ± 0.71 ^h–o^	1.50 ± 0.23 ^c–j^
	Y5	2.27 ± 0.80 ^m–o^	1.01 ± 0.70 ^f–j^
	Y6	1.60 ± 0.43 ^o^	0.64 ± 0.26 ^h–j^
	Y7	3.83 ± 0.56 ^g–n^	2.04 ± 0.39 ^b–j^
	Y8	3.15 ± 0.57 ^h–o^	1.41 ± 0.29 ^c–j^
	Y9	2.87 ± 0.87 ^j–o^	0.67 ± 0.75 ^h–j^
	Y10	3.27 ± 0.76 ^h–o^	0.97 ± 0.85 ^f–j^
	Y11	5.64 ± 1.38 ^d–g^	1.38 ± 0.86 ^d–j^
	Y12	4.18 ± 0.64 ^f–m^	2.43 ± 0.59 ^b–i^
	Y13	2.64 ± 0.63 ^k–o^	1.12 ± 0.27 ^e–j^
	Y14	5.52 ± 1.71 ^d–g^	3.10 ± 0.40 ^b–d^
	Y15	3.22 ± 0.59 ^h–o^	2.30 ± 0.27 ^b–i^
	Y16	2.76 ± 0.42 ^k–o^	1.38 ± 0.21 ^d–j^
	Y17	4.51 ± 1.16 ^e–k^	2.00 ± 2.19 ^b–j^
	Y18	4.53 ± 1.08 ^e–k^	2.29 ± 2.52 ^b–i^
	Y19	6.25 ± 0.93 ^c–e^	3.41 ± 2.16 ^b^
	Range	1.60–6.25	0.64–3.41
	Mean	3.48	1.64
Black	B1	4.49 ± 0.67 ^e–k^	2.40 ± 0.25 ^b–i^
	B2	2.01 ± 0.60 ^n–o^	0.88 ± 0.16 ^g–j^
	B3	1.83 ± 0.77 ^o^	0.28 ± 0.39 ^j^
	B4	2.49 ± 0.58 ^l–o^	0.83 ± 0.27 ^g–j^
	B5	2.76 ± 1.27 ^k–o^	0.58 ± 0.62 ^i–j^
	B6	4.11 ± 0.66 ^f–m^	1.80 ± 0.38 ^b–j^
	B7	2.35 ± 0.19 ^l–o^	1.02 ± 0.37 ^f–j^
	B8	4.02 ± 0.31 ^f–m^	2.27 ± 0.43 ^b–i^
	B9	3.21 ± 0.82 ^h–o^	1.35 ± 031 ^d–j^
	B10	4.08 ± 0.65 ^f–m^	1.96 ± 0.24 ^b–j^
	B11	5.23 ± 1.06 ^d–g^	2.35 ± 0.40 ^b–i^
	B12	2.93 ± 0.81 ^i–o^	1.66 ± 0.69 ^b–j^
	B13	4.78 ± 0.71 ^e–j^	1.71 ± 0.43 ^b–j^
	B14	4.87 ± 1.22 ^e–i^	1.25 ± 0.99 ^d–j^
	B15	8.34 ± 1.30 ^b^	3.02 ± 1.04 ^b–e^
	B16	4.26 ± 1.38 ^f–l^	0.77 ± 0.43 ^g–j^
	B17	11.93 ± 1.08 ^a^	6.20 ± 0.35 ^a^
	B18	4.82 ± 0.63 ^e–i^	2.84 ± 0.60 ^b–f^
	B19	3.82 ± 0.63 ^g–n^	1.79 ± 0.35 ^b–j^
	B20	7.42 ± 1.76 ^bc^	3.53 ± 0.72 ^b^
	B21	5.78 ± 1.11 ^c–f^	2.22 ± 1.98 ^b–i^
	B22	4.71 ± 1.24 ^e–j^	1.31 ± 1.96 ^d–j^
	B23	7.98 ± 1.49 ^b^	3.42 ± 2.16 ^b^
	B24	6.77 ± 1.03 ^b–d^	3.29 ± 1.22 ^bc^
	B25	4.71 ± 0.96 ^e–j^	2.57 ± 0.72 ^b–h^
	Range	1.83–11.93	0.28–6.20
	Mean	4.79	2.05
Green–Black	G1	2.39 ± 1.08 ^l–o^	1.38 ± 0.14 ^d–j^
	G2	1.91 ± 0.92 ^n–o^	0.67 ± 0.96 ^h–j^
	G3	2.71 ± 0.45 ^k–o^	1.48 ± 0.65 ^c–j^
	G4	5.66 ± 1.29 ^d–g^	2.63 ± 1.80 ^b–g^
	Range	1.91–5.66	0.67–2.63
	Mean	3.17	1.54

^a–o^ Means followed by the same letter within rows are significantly different at *p* < 0.05, Duncan’s multiple range test.

**Table 3:** GWAS of 48 individual soybean isoflavone derivatives. ABTS (radical scavenging activity), DPPH (radical scavenging activity), estrogen (estrogen activity), ER-α (estrogen receptor alpha activity), UCP-1 (uncoupling protein-1 activity), NO (nitric oxide inhibition activity).

Traits	Count					
	ABTS	DPPH	Estrogen	ER-α	UCP-1	NO
Genistein 7-O-(2″-O-apiosyl)glucoside	67	22	33	73	66	42
Genistein 7-O-(6″-O-acetyl)glucoside (6″-O-acetylgenistin)	4	2	29	21	43	14
Glycitein	36	30	113	367	185	209
Total	107	54	175	461	294	265

## Data Availability

Data is contained within the article.
